# Influence of the Physical Characteristics of Feed on the Digestive Processes of Atlantic Salmon, *Salmo salar*, Focusing on Gut Transit Time

**DOI:** 10.1155/anu/3269414

**Published:** 2026-01-18

**Authors:** Paige C. Miles, Thomas S. Mock, Matthew K. Jago, Michael J. Salini, Richard P. Smullen, David S. Francis

**Affiliations:** ^1^ Nutrition and Seafood Laboratory, Deakin University, Queenscliff, Victoria, Australia, deakin.edu.au; ^2^ Ridley AgriProducts Pty Ltd, Narangba, Queensland, Australia

**Keywords:** aquaculture, aquafeed, digestion, gastrointestinal tract

## Abstract

Rearing variables, such as water temperature and dietary composition, can significantly affect gut transit time (GTT) in cultured Atlantic salmon (*Salmo salar*). These influences can accelerate the transit of digesta, leading to reduced nutrient digestibility and uptake, whilst impacting feed intake, appetite and gut evacuation, which collectively may result in inefficient feed utilisation in commercial aquaculture. To address these issues, manipulating the physical properties of the diet, specifically pellet and raw material particle size, may present a solution via the modulation of the GTT in fish. This study aimed to evaluate the GTT of 360 pre‐smolt Atlantic salmon (120.58 ± 0.25 g) fed one of six dietary treatments varying in raw material particle size (fine: 0–250 μm, medium: 250–500 μm or coarse: 500 μm–1000 μm) and subsequent pellet size (3 mm or 5 mm) using a factorial design. GTT was quantified by assessing the relative distribution of an inert feed marker (ballotini® beads), incorporated in the feed, in three defined digestive tract regions: the stomach (ST), mid‐intestine (MI) and distal intestine (DI). It was found that the larger pellet size resulted in faster transit of digesta, but the relationship between raw material particle size (fine, medium or coarse) and transit rate was highly variable. However, the time taken for 50% evacuation of the digesta present in the GI tract is modulated by both pellet and raw material particle size. The insights gained from this research could guide the development of commercial diets designed to mitigate the negative impacts of an accelerated gut transit. Consequently, this approach has the potential to enhance feed efficiency from both nutritional and economic perspectives and reduce the environmental impact caused by excessive nitrogenous waste production in aquaculture.

## 1. Introduction

Gut transit time (GTT), the time required for digesta to pass through the gastrointestinal tract (GIT) from the point of feed ingestion to the excretion of waste, is an integral digestive process that is intricately linked to nutrition and health in cultured monogastric species such as Atlantic salmon (*Salmo salar*) [1–3]. GTT influences nutrient uptake by dictating the time available for the exposure of digesta to the digestive enzymes present in the GIT [1], particularly within the mid‐intestine (MI), which represents a primary site for nutrient absorption [2, 4, 5]. As such, accelerated GTT, in part, can influence the assimilation of dietary nutrients in cultured species, by limiting the time available for sufficient nutrient absorption, as would be experienced with a slower GTT [2, 3, 6]. Additionally, GTT is closely linked to feeding behaviour, influencing the return of appetite, hunger and satiety cues and feed intake in numerous fish species [7–10]. In addition to the regulation of appetite and intake via the actions of orexigenic and anorexigenic neurones, hunger in fish is largely influenced by the amount of partially digested feed present in the stomach (ST) [9, 11]. As the ST empties, fish experience hunger, and overtime, appetite returns [9, 10]. Whilst the release of appetite regulating hormones are well‐documented and undoubtedly essential in the process of hunger and subsequent intake, the physical process of gastric emptying warrants further exploration [12, 13]. Gaining an increased understanding of GTT in farmed fish may permit the development of more precise feeding schedules and more judicious dietary formulations in intensive aquaculture settings.

Past studies have found that GTT in Atlantic salmon can be influenced by culture variables, including rearing temperature [3], dietary formulation [14] and pellet physical properties [14]. Following prolonged exposure to water temperatures of 18–20°C, Atlantic salmon were shown to demonstrate significantly faster GTT in comparison to fish reared at 10–14°C [3]. With respect to diet, Sveier et al. [14] observed that GTT was faster in Atlantic salmon fed a diet in which the raw materials were processed at a finer grind size (100–300 μm). Similarly, Busti et al. [15] determined that gilthead sea bream (*Sparus aurata*) fed a 2 or 4 mm pellet resulted in a faster gastric emptying rate than those conditioned to a 6 mm pellet. Short‐term and rapid GTT may have positive outcomes in fish, including improved feed intake and growth [16], however, long‐term and accelerated GTT may have negative impacts. The influence of increased GTT on nutrient absorption can result in the inefficient use of dietary nutrients by restricting the time available for assimilation, regardless of the digestibility of the individual raw materials used in the dietary formulation [1]. Additionally, GTT influences the feeding behaviour of fish and can result in a reduced interest in feeding, or the expression of hunger signals that do not correlate to scheduled feeding events [9, 10, 17]. Collectively, increased GTT can cause significant issues from an environmental perspective, where waste generated from uneaten feed and poorly digested feed leads to greater levels of nitrogen‐ and phosphorus‐rich waste entering the environment surrounding aquaculture operations [18, 19]. These inputs can lead to a series of issues including eutrophication [20], increased turbidity [21] and deterioration of the benthic zone [22]. Taken together, the negative biological, environmental and economic repercussions associated with prolonged accelerated GTT, necessitates a greater understanding of the factors impacting GTT and the exploration of viable solutions for mitigating the issue are of particular interest.

Once feed pellets are ingested and broken down into their constituent particles, they move through the GIT at varying rates depending on the size of the particle and the extent to which the particles are indigestible or refractory [23–25]. In comparison to larger counterparts, smaller particles are understood to facilitate rapid, physical movement of digesta that is less likely to accumulate in the GIT [24, 26]. The identified mechanisms that drive the observed relationships between feed pellet size and GTT are moisturisation and peristaltic activity [23]. Specifically, a physically larger sized pellet requires a greater amount of time to moisturise and macerate following ingestion, subsequently impacting the transit of digesta to the latter regions of the GIT in comparison to what would be observed with a smaller pellet size. Whilst various studies have explored the links between pellet size or raw material particle size and GTT in farmed species, the resultant impacts on nutrient digestibility remain under‐explored. As such, the aims of this study are to assess the effect that ingredient particle and pellet size can have on the GTT of Atlantic salmon and the subsequent impact on nutrient digestibility. It is anticipated that these findings may inform the development of diets with the goal of optimising the efficient utilisation of dietary nutrients through a better understanding of the effects of GTT in fish.

## 2. Materials and Methods

### 2.1. Raw Material Particle Size Distribution of the Protein Sources

To achieve three distinct feed raw material particle size ranges for the experimental diets (0–250 μm, 250–500 μm or 500–1000 μm), the main protein sources of the diet formulation: lupin meal (LP), chicken protein concentrate (CPC), fish meal (FM) and soy protein concentrate (SPC), were milled to <1000 μm and sieved to the distinct raw material particle size ranges. The particle size distribution of both the raw ingredients and a sample (Figure 1) of the dry diet mash, were subject to a particle size distribution test using an automated sieve shaker (Endecotts, London, United Kingdom). Following this analysis, each sieve pan was weighed to determine the amount of sample in the corresponding particle size range. The known pan weight was subtracted, allowing for the calculation of a known sample weight. The sample weight was then used to determine relative raw material particle size distribution using the following formula:

Figure 1Images of the three dry basal mash formulations containing lupin meal (LP), chicken protein concentrate (CPC), fish meal (FM) and soy protein concentrate (SPC) sieved to a (a) fine (0–250 μm), (b) medium (250–500 μm) or (c) coarse (500–1000 μm) size class to create diets of differential raw material particle size distribution, prior to being pelletised.

**Figure:**
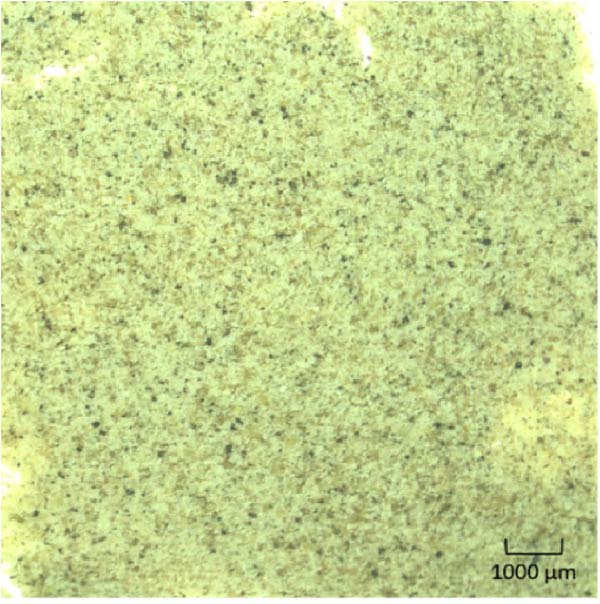
(a)

**Figure:**
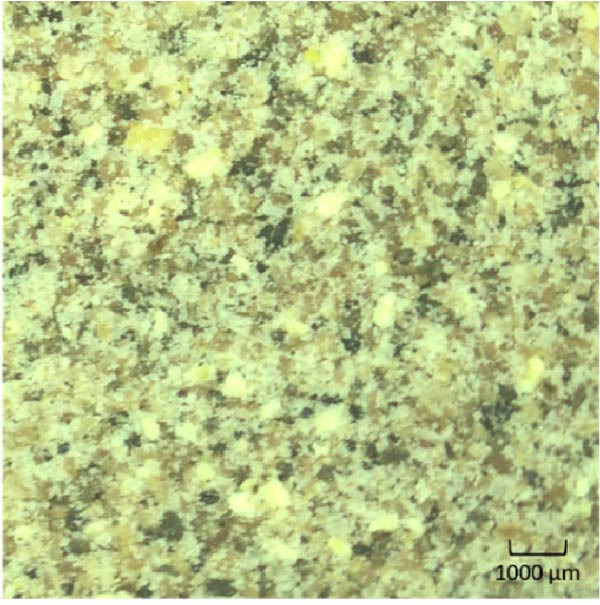
(b)

**Figure:**
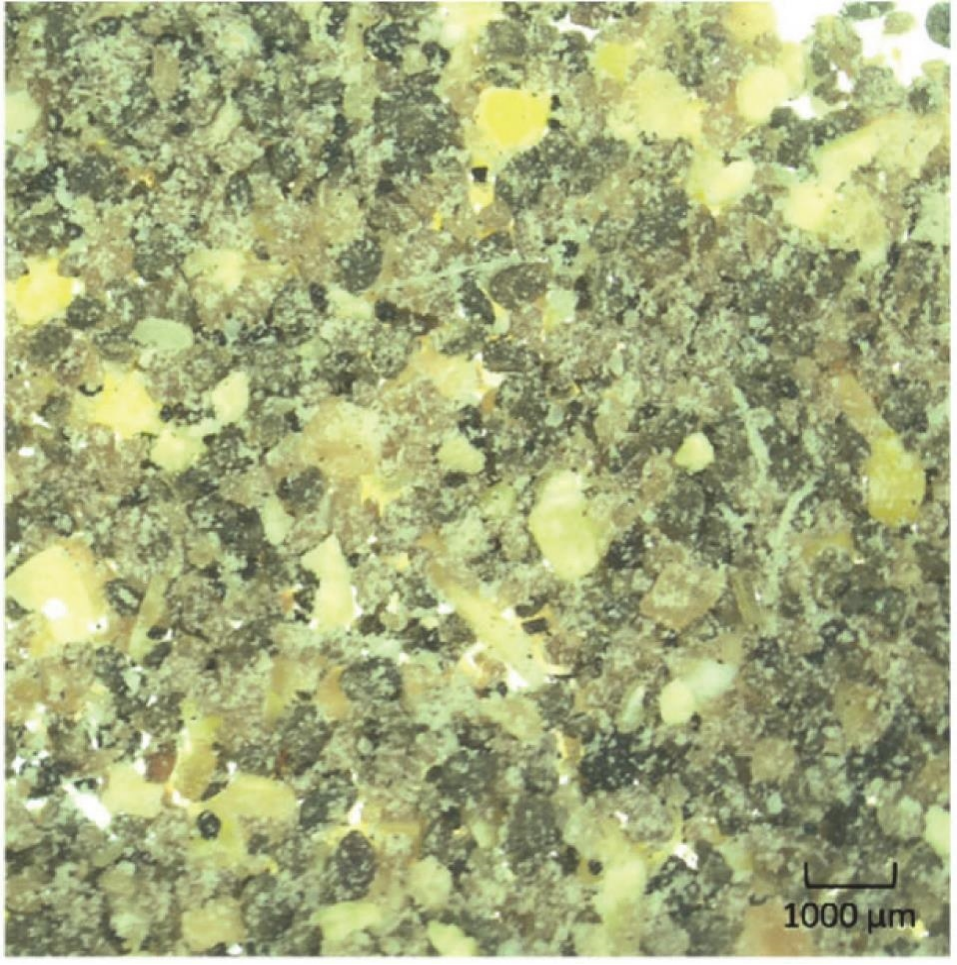
(c)

Relative feed ingredient particle size distribution %=Sample weight in sieve pan gTotal sample weight (g)×100.

The analysis determined the raw material particle size distribution of the four main protein sources as received from the supplier (Ridley Agriproducts Pty Ltd, Australia) and ensured that the sieving process achieved the target particle distribution of each dietary formulation. Blood meal and wheat gluten were not prepared by size class separation in this experiment as the grind size was, in general, too fine to achieve the target raw material particle size classes.

### 2.2. Diet Preparation and Formulation

Six isolipidic, isoproteic and isoenergetic diets were formulated for this study (Table 1). The formulation of the diets was identical, varying only in the raw material particle size distribution of the protein sources and the size of the extruded pellet. All diets were formulated to meet the nutritional requirements of juvenile, pre‐smolt Atlantic salmon [27]. The diets were produced in two pellet sizes, using either a 3 or 5 mm aperture die, using the sieved protein sources from each of three feed raw material particle size classes: 0–250 μm (fine), 250–500 μm (medium) or 500–1000 μm (course). The protein ingredients sieved to distinct particle size ranges contributed to 50% of ingredients (weight) of the total diet mash. The diets were referred to as: 3 mm: fine, 3 mm: medium, 3 mm: coarse, 5 mm: fine, 5 mm: medium and 5 mm: coarse. A 25 kg basal mash of each diet was prepared, to which the sieved ingredients were incorporated to create the treatments, then mixed using a commercial spiral mixer (MEC Australia, Preston, Victoria, Australia). When a homogenous mix was achieved, water (25%) and the dietary lipid sources were added until a dough‐like consistency was achieved. Subsequently, 1.5 kg of each complete mash was removed, and a 0.5% inclusion of an inert GTT marker (ballotini® beads, 425–600 μm) was added to the 1.5 kg aliquot and mixed thoroughly until an even distribution of the beads throughout the mash was achieved to avoid transit bias in the GIT. Each of the beaded and un‐beaded batches of mash were extruded separately into 3 and 5 mm pellets using a single screw extruder at a temperature of approximately 80°C (Shanghai Tolcat Industrial Co, Ltd, China) as per Miles et al. [28] to achieve consistent sizing of the finished product [29]. The extrusion conditions, namely, pressure and temperature, were kept constant during the manufacturing of the experimental diets. Once extruded, pellets were dried for 48 h at 40°C, until a consistent moisture content was achieved. All diets were stored in airtight containers until the commencement of the trial. The non‐beaded diets were made for use through the duration of the trial phase, and the corresponding beaded versions of each diet were made to be used during one single feeding event at the end of the trial as described in detail later.

**Table 1:** Diet formulation (%) of the reference and beaded formulation used for each of the six experimental diets: 3 mm: fine, 3 mm: medium, 3 mm: coarse, 5 mm: fine, 5 mm: medium and 5 mm: coarse, fed to Atlantic salmon to assess the modulation capabilities of two pellet and three raw material particle sizes.

Ingredient	Inclusion level (%)
Lupin meal^a^	5
Fish meal^a^	20
Chicken protein concentrate^a^	20
Soy protein concentrate^a^	5
Blood meal	5
Wheat flour	19.58
Wheat gluten	7.5
Poultry oil	11
Fish oil	4.53
Vitamin C (stabilised)	0.22
Vitamin E	0.02
Choline chloride (70%)	0.09
DL‐methionine	0.14
Vitamin and mineral premix^b^	0.85
Astaxanthin (10%)	0.05
Monocalcium phosphate	0.97
Yttrium oxide^c^	0.05
Total	100

^a^Raw protein sources that were sieved to each of the following raw material particle sizes: 0–250 μm, 250–500 μm and 500 μm–1 mm.

^b^Commercially available vitamin and mineral premix provided by Ridley Agriproducts.

^c^Inert dietary marker used to assess digestibility.

The physical pellet properties of hardness and bulk density were assessed on 20 samples of each of the six experimental diets (Table 2). Pellet hardness (g F) was determined using a force gauge (Mark‐10, New York, United States of America) press that determines the resistance of the pellet to breaking under force. Pellet bulk density was determined by filling a container of a known volume with a dietary treatment, ensuring that the top of the container was levelled. The filled container was placed on a tared scale and the weight recorded (g). The known weight was divided by the volume of the container (mL) to calculate bulk pellet density expressed as g cm^−3^.

**Table 2:** Proximate composition and physical pellet properties of the six experimental diets (unbeaded) fed to Atlantic salmon to assess the modulation capabilities of two pellet and three raw material particle sizes.

	Dietary treatment					
	3 mm: fine	3 mm: medium	3 mm: coarse	5 mm: fine	5 mm: medium	5 mm: coarse
Diet proximate composition (g kg^−1^)^a^						
Moisture	56.9	72.4	70.4	67.0	49.3	64.6
Crude protein	461.1	454.4	458.1	454.6	461.8	456.2
Total lipid	211.0	203.2	204.6	207.5	204.5	207.2
Ash	55.2	54.4	50.0	55.2	55.8	60.2
Gross energy (kJ kg^−1^)^b^	22.9	22.5	22.6	22.6	22.9	22.5
Pellet hardness (g F)	668.1	512.1	353.3	801.5	691.1	377.2
Bulk pellet density (g cm^−3^)	0.55	0.57	0.49	0.50	0.44	0.47

*Note:* Values are expressed as the mean (*n* = 3, *N* = 18).

^a^Calculated on an as fed basis.

^b^Gross energy calculated using standard factors of 23.6, 39.5 and 17.2 MJ kg^−1^ for protein, fat and carbohydrate, respectively [27].

### 2.3. Experimental Design and Animal Husbandry

All animals and procedures utilised throughout the study were approved by the Deakin University Animal Ethics Committee (Application Number B17‐2022).

The experiment was conducted at the Deakin Aquaculture Futures Facility (DAFF; Warrnambool, Victoria, Australia). Atlantic salmon utilised in this study were sourced from Mountain Fresh Trout and Salmon Farm (Harrietville, Victoria, Australia). Prior to the trial phase, fish were acclimated for 14 days under experimental conditions. Fish were reared in a freshwater, closed‐loop and recirculating aquaculture system with photoperiod and thermostatic control, comprising of 18 × 1000 L tanks. Water quality was maintained via UV disinfection and biological and physical filtration systems (Drumscreen filter fitted with a 60‐μm screen; Hydrotech, Vellinge, Sweden). Total ammonia (<5 mg L^−1^), nitrite (<5 mg L^−1^) and nitrate (<100 mg L^−1^) were monitored daily and maintained well below acceptable limits. Water temperature was maintained at 15.0 ± 0.5°C for both the acclimation and trial phase. Once acclimated, 360 Atlantic salmon with an average weight of 120.58 ± 0.25 g were weighed in groups of five fish and randomly distributed between the 18 experimental tanks (20 fish per tank; average tank biomass = 2411.67 ± 4.95 g). Three replicate tanks were assigned to each of the six dietary treatments at random. During the trial phase (35 days), fish were fed to satiety twice daily at 0900 and 1700 h. Satiation was assessed visually by experienced feeders. After 14 days of the trial phased, fish were anaesthetised 15 h after feeding in an AQUI‐s solution (~20 mg L^−1^ of a stock solution of isoeugenol 540 mg L^−1^, Lower Hutt, New Zealand) as per manufacturer instructions, weighed and faeces samples were collected from all fish (20 fish) via manual stripping by applying gentle pressure on the abdominal region of the fish until a sufficient amount of faecal matter was collected, pooled by tank, for subsequent determination of nutrient digestibility. Following faecal collection, fish remained in the tanks, being fed their respective experimental diets for the remainder of the trial phase (21 days).

### 2.4. Feed Efficiency and Growth Performance Calculations

Data pertaining to growth and feed intake were generated using the formulae below to ensure that the dietary treatments did not cause any adverse effects. Calculations included weight gain (total and %), specific growth rate (SGR%/day), feed conversion ratio (FCR) and specific feed rate (SFR):

Total weight gain g=Final weight g−initial weight g.

Weight gain %=100×Final weight g−initial weight gInitial weight g.

Specific growth rate=100×lnfinal weightg−lninitial weightgTime days.

Feed conversion ratio =Feed intake gWeight gain g.

Specific feeding rate =100×Feed intakegAverage fish weight g.

### 2.5. Proximate Composition Analysis

Proximate composition of the experimental diets (duplicate; Table 2) was determined following procedures outlined by Lewis et al. [30]. In brief, moisture was determined by drying in an oven at 80°C overnight to a constant weight. Similarly, ash content was determined by incinerating samples in a muffle furnace (Labec Laboratory Equipment Pty Ltd, Australia) at 550°C for 18 h. Protein (Kjeldahl nitrogen; *N* x 6.25) was determined via the Kjeldahl method [31] using an automated Kjeltech 8400 (FOSS, Analytical Co. Ltd, Sweden). Total lipid content was determined via modified dichloromethane: methanol extraction (2:1) as described by Folch et al. [32]. Total energy was calculated on the basis of known combustion enthalpies of 23.6, 39.5 and 17.2 kJ g^−1^ for protein, lipid and carbohydrate, respectively [27].

### 2.6. Nutrient Digestibility

The apparent digestibility of the dry matter, macronutrients and gross energy of the diets (duplicate) were determined using faecal samples pooled by tank (*N =* 18) obtained 3 weeks prior to fish sampling for GTT analysis and samples of each of the six experimental diets, following methods outlined by Rocker et al. [33]. In brief, inductively coupled plasma mass spectroscopy (ICPMS) was used to determine the concentration of the internal marker (yttrium oxide) in feed and faeces samples. Samples were analysed at the Plant Chemistry Research Laboratory (School of Life and Environmental Sciences, Deakin University, Burwood Campus). Diet and faeces samples were assessed in triplicate, and results were reported as the mean ± standard error (SE). Apparent digestibility coefficients (ADCs) were calculated following the following formulae:

ADC of dry matter %=100100−dietary markerfaecal marker×.

ADC of nutrients or energy %=1100−dietary markerfaecal marker×faecal nutrient or energydetary nutrient or energy×.

### 2.7. Bead Administration and Retrieval

Immediately preceding the first GTT sampling time point, fish were fed a single set ration of 10 g kg^−1^ body weight of the beaded form of their allocated diet informed by satiety feed intake data obtained two weeks prior to the sampling event (Table 1). An inert beaded marker was selected for this study to allow the assessment of the transit of digesta through the individual GIT regions [28]. Furthermore, the fine size of the beads utilised in this study (425–600 μm) allows for the transit of the marker at a rate relative to the surrounding digesta, providing an accurate depiction of GTT. Simultaneously, the authors acknowledge that the small inclusion of the inert beaded marker may have had subtle effects on GTT, yet the inclusion rate of the beads were the same across all diets. Therefore, this is not expected to hamper the interpretation of the results. Feeding of the beaded formula commenced at 0900 h (time‐point: ‘0’), concluding at 0940. A staggered feeding approach was employed where tanks were fed the ration simultaneously in groups of six, with a time frame of 10 min being allocated to each feeding period. Staggered feeding was conducted to avoid overlap at the time of sampling. As such, the sampling of each tank reflected the time elapsed since feeding, that is, 2 h post‐feed. Sampling of fish from each tank occurred periodically at seven distinct time points: 2, 4, 8, 16, 24, 36 and 48 h post‐feed. At each time point, with the exception of the final time point where only two fish remained in the tank, three fish from each tank, were humanely killed using an overdose (100 mg L^−1^) of AQUI‐S anaesthetic (isoeugenol 540 mg L^−1^). A repeat tank interaction sampling design was adopted to minimise the number of fish required to conduct this study. Past studies adopting the same strategy confirm the robustness of the GTT data and did not impart a stress response in fish [28]. All fish sampled during the sampling period were placed in labelled freezer bags and stored at −20°C in preparation for future analysis of GTT.

Relative bead distribution was determined by retrieving the inert ballotini® bead consumed by the fish in this study from the dissected GIT. Frozen GIT, with the pyloric caeca and adipose tissue removed, were stored at −20°C following dissection as per previously described protocols [3] pending analysis. The GIT was then separated into three distinct sections as defined by Mock et al. [3], henceforth, referred to for brevity as: the ST, the MI and the distal intestine (DI). Once separated, the tract regions were placed into individual, labelled crucibles and incinerated in a muffle furnace (Tetlow, Victoria, Australia) at 550°C for 18 h. The samples were then individually rinsed with water through a 250 μm metal sieve to remove the remaining inorganic matter. Any large particles that remained were gently pushed through the sieve using a silicone spatula or a laboratory wash bottle containing distilled water. Sieves containing what remained of the sample were dried in an oven (Model: UM300, Memmert GmbH, Germany) at 120°C to evaporate any residual moisture. When dry, sieves were removed from the oven, and the remaining sample was gently transferred onto a microscope slide that had been primed with glycerine, serving as a substrate to suspend the remaining beads on the slide. A microscopic image was taken using a Stemi 508 with an Axiocam attachment (Carl Zeiss Microscopy GmbH, Germany) and generated in the programme Zen 3.0 (Blue Edition; Carl Zeiss Microscopy GmbH, Germany). These images were saved as a JPEG file in preparation for bead quantification.

### 2.8. Bead Quantification

Images of ballotini® beads retrieved from the separate regions of the GIT were processed in Adobe Photoshop as per [28]. The number of beads present in each sample was quantified using the counting tool function in ImageJ. Bead counts from this process were then collated to determine the relative feed distribution. GTT was informed by the bead density (%) in an individual GIT section relative to the total number of beads in the entire GIT, quantified using the following formula:

Feed distribution in the GIT % =Ballotini beads in X at time tTotal number ballotini beads in Y at time t×100,

where *X* is equal to the GIT region (ST, MI or DI) and *Y* is the entire GIT and *t* is the time.

The amount of feed evacuated for the GIT was calculated for each time point. This was expressed as a proportion (%) of the ingested feed and was calculated using the following formula:

Gut evacuation of ingested feed %= ∑Ballotini beads in X at time tTotal number ballotini beads in Y at time 2× 100,

where *X* is equal to the GIT region (ST, MI or DI) and *Y* is the entire GIT, *t* is the time and time 2 is 2 h post‐feed. Following this, linear equations were used to plot the gut evacuation data. Additionally, the time at which 50% (*T*_50%_) of ingested feed had evacuated the entire GIT was used as a point of comparison between treatments by solving these linear equations, similar to a previous study by Sveier et al. [14], who instead evaluated ST evacuation.

### 2.9. Statistical Analysis

All data and figures were reported as mean ± SE. Statistical analyses presented herein were performed using R‐Studio (Ver. 4.2.1). Differences in weight gain, final weight, FCR, SFR and SGR data were subject to a two‐way analysis of variance (ANOVA). Similarly, ADC of diets across dietary treatments were analysed using two‐way ANOVA implemented through generalised linear models (GLMs). A multi‐way ANOVA was conducted to assess differences in relative bead density between the GIT regions between treatments both across time, and to identify interactive effects between time × treatment, if present. Following, differences in relative bead density in the GIT regions between treatments and within time points were determined via a two‐way ANOVA. A *T*‐test or Tukey’s post hoc test were performed to identify significant differences within pellet sizes or raw material particle sizes, respectively. *T*_50%_ values, determined by solving linear equations used to plot total gut evacuation, were compared between treatments.

## 3. Results

### 3.1. Proximate Composition and Physical Characteristics of Diets

All dietary treatments utilised in this study, in both the beaded and un‐beaded form were well accepted by all fish. Assessment of proximate composition (Table 2) of the six dietary treatments determined that the diets were isolipidic (~200 g kg^−1^), isoproteic (~460 g kg^−1^) and isoenergetic (~22 kJ g^−1^), as per the intended formulation. Pellet hardness ranged between 353.25 g F (3 mm: coarse) and 801.45 g F (5 mm: fine; Table 2). Bulk pellet density values ranged between 0.44 and 0.57 g cm^−3^ for the 5 mm: medium and 3 mm: coarse treatments, respectively.

### 3.2. Growth Performance Metrics

Growth performance metrics including initial weight, weight gain, final weight, FCR, SFR and SGR are reported in Table 3. Average initial weight across all treatments at the time of allocation was 120.6 ± 0.3 g. Average final weight recorded at the conclusion of the trial was 216.3 ± 2.4 g, resulting in a weight gain of 79.4 ± 2.0%. There were no statistically significant differences between the six dietary treatments across the assessed growth or feed performance metrics. The average reported values for FCR, SFR and SGR were 1.00 ± 0.02, 1.15 ± 0.02 and 1.19 ± 0.02, respectively.

**Table 3:** Initial weight, weight gain (total and %), final weight, FCR, SFR and SGR of Atlantic salmon fed one of six dietary treatments assessing two pellet size and three raw material particle sizes for 36 days at 15°C.

	Dietary treatment						*p*‐Value
	3 mm: fine	3 mm: medium	3 mm: coarse	5 mm: fine	5 mm: medium	5 mm: coarse	
Initial weight (g)	120.7 ± 0.5	120.7 ± 0.7	121 ± 0.3	120.5 ± 1.0	119.9 ± 1.0	120.7 ± 0.4	0.930
Final weight (g)	216.7 ± 9.0	215 ± 7.0	221.2 ± 6.9	222.3 ± 6.2	207.9 ± 2.5	214.5 ± 2.9	0.634
Weight gain (g)	96.0 ± 9.4	94.3 ± 6.4	100.2 ± 7.2	101.8 ± 6.5	88.0 ± 3.5	93.8 ± 3.2	0.703
Weight gain (%)	79.5 ± 8.1	78 ± 4.9	82.9 ± 6.1	84.5 ± 5.6	73.5 ± 3.5	77.8 ± 2.8	0.754
FCR	1.02 ± 0.08	0.98 ± 0.04	0.96 ± 0.04	0.95 ± 0.03	1.1 ± 0.03	0.98 ± 0.06	0.389
SFR	1.17 ± 0.01	1.13 ± 0.07	1.15 ± 0.04	1.15 ± 0.03	1.21 ± 0.05	1.11 ± 0.04	0.752
SGR	1.19 ± 0.09	1.18 ± 0.06	1.23 ± 0.07	1.25 ± 0.06	1.12 ± 0.04	1.17 ± 0.03	0.762

*Note:* Values are expressed as the mean ± SE (*n* = 3, *N* = 18). ANOVA with post hoc Tukey’s test.

### 3.3. Nutrient Digestibility

ADCs (ADC %) of the six experimental diets are reported in Table 4. The ADC of dry matter ranged between 65.6 ± 0.5 and 68.4 ± 0.5 in the coarse (pooling raw material particle size) and fine (pooling raw material particle size) treatments, respectively (*p*  < 0.05). There were no significant differences between the ADC of protein, lipid and gross energy of the pellet sizes (pooling raw material particle size). Protein digestibility was statistically higher in the 3 mm pellets (pooling raw material particle size; 88.8 ± 0.3) than the 5 mm treatments (88.0 ± 0.2). Assessment of raw material particle size (pooling pellet size) determined that the fine (85.6 ± 0.4) and medium (85.5 ± 0.4) raw material particle size treatments recorded significantly higher gross energy digestibility compared to the coarse treatment (83.8 ± 0.4). Results pertaining to the ADC of protein and lipid were not significantly different between the raw material particle sizes (pooling pellet size). ADC values pertaining to energy ranged between 83.5 ± 0.5 and 86.3 ± 0.3 in the 5 mm: coarse and 3 mm: fine treatments, respectively (*p*  < 0.05). All other ADC parameters measured presented no significant differences in response to the treatments.

**Table 4:** Apparent digestibility coefficients (ADC%) of the six experimental diets assessing two pellet size and three ingredient particle fed to Atlantic salmon reared at 15°C for 36 days.

Dietary treatment							Pellet		Ingredient particle			*p*‐Value		
	3 mm: fine	3 mm: medium	3 mm: coarse	5 mm: fine	5 mm: medium	5 mm: coarse	3 mm	5 mm	Fine	Medium	Coarse	Pellet (A)	Ingredient particle (B)	A × B
Dry matter	69.4 ± 0.5	66.6 ± 1.8	65.9 ± 0.5	67.4 ± 0.3	67.6 ± 0.3	65.4 ± 0.8	67.3 ± 0.8	66.8 ± 0.4	68.4 ± 0.5a	67.1 ± 0.8^ab^	65.6 ± 0.5^b^	ns	^∗^	ns
Protein	88.9 ± 0.1	88.9 ± 0.8	88.5 ± 0.3	87.6 ± 0.3	88.5 ± 0.3	87.9 ± 0.5	88.8 ± 0.3^a^	88.0 ± 0.2^b^	88.2 ± 0.3	88.7 ± 0.4	88.2 ± 0.3	^∗^	ns	ns
Lipid	92.7 ± 0.1	92.2 ± 0.6	91.4 ± 0.3	92.0 ± 0.3	92.5 ± 0.4	91.7 ± 0.3	92.1 ± 0.3	92.1 ± 0.2	92.4 ± 0.2	92.3 ± 0.3	91.6 ± 0.2	ns	ns	ns
Gross energy	86.3 ± 0.3^a^	85.6 ± 0.8^ab^	84.2 ± 0.8^ab^	85.0 ± 0.5^ab^	85.3 ± 0.4^ab^	83.5 ± 0.3^b^	85.3 ± 0.5	84.6 ± 0.3	85.6 ± 0.4^a^	85.5 ± 0.4^a^	83.8 ± 0.4^b^	ns	^∗^	^∗^

*Note:* Values are expressed as the mean ± SE (*n* = 3, *N* = 18). ANOVA with TT‐test (pellet) or post hoc Tukey’s test (dietary treatment or ingredient particle). Different superscript letters indicate significant differences (*p* < 0.05) between the means of different treatments.

^∗^Denotes significance (*p*  < 0.05).

### 3.4. Relative Bead Density in the GIT

**Figure 2:**
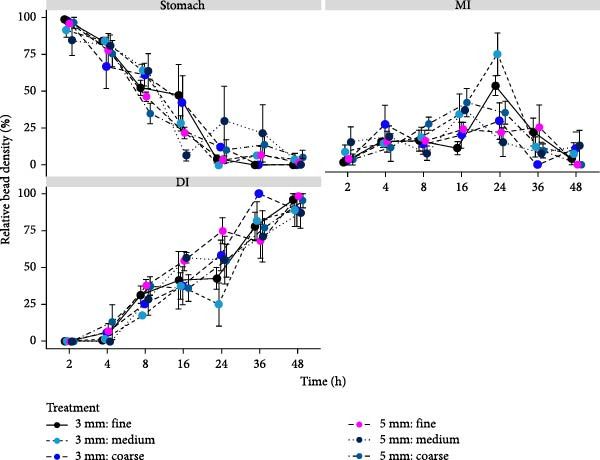
Relative bead distribution ± SE (*n* = 3, *N* = 18) in the ST, MI and DI of Atlantic salmon reared at 15°C fed one of six dietary treatments assessing two pellet size and three raw material particle sizes for 36 days. Relative bead density was quantified by determining the relative density of beads detected in each region relative to the total number of beads found in the GIT at the time of sampling. Relative bead density (%) is presented on the *Y*‐axis and time post‐feed (hours) is presented on the *X*‐axis.

The relationship between relative bead density in the GIT and the effect of ingredient particle and pellet size over 48 h following feeding of Atlantic salmon was determined by assessing the relative distribution of ballotini beads across the three chosen regions of the GIT: the ST, MI and DI, relative to the total number of beads present in the GIT across seven distinct time points (2, 4, 8, 16, 24, 36 or 48 h post‐feed; Figure 2). Time had a significant effect on the relative bead density in all regions of the GIT (*p*  < 0.05). Furthermore, a significant time × pellet interaction was recorded in the ST (*p*  < 0.05; Table 5). In the ST, there was an overall negative relationship between relative bead distribution and time regardless of dietary treatment. The maximum relative bead density observed in the ST was at 2 h post‐feed in the 3 mm: fine treatment (98.4 ± 1.6%; Table 6). The clear negative relationship between relative bead density and time in the ST was inverse to a strong positive relationship in the DI. The maximum bead density recorded in the DI was 100.0 ± 0.0% and occurred at 36 h post‐feed in the 3 mm: coarse treatment. Whilst there was a significant effect of time on the relative bead distribution in the MI, the variability between time points in the MI resulted in no obvious discernible positive or negative trends. The maximum relative bead density detected in the MI occurred at 24 h post‐feed in the 3 mm: fine treatment (75.0 ± 14.4%). Following the assessment of the relationship between particle and pellet size, and the relative bead density of each GIT region across time points, differences in bead density between treatments was assessed within time points (Table 6). A single significant effect of treatment was detected in the MI at 24 h post‐feed. Values ranged between 22.0 ± 7.2% (3 mm: coarse) and 75.0 ± 14.4% (3 mm: medium). For variables where there were no significant interactions between particle and raw material particle size, the effect of raw material particle size (pooling pellet size) or pellet size (pooling raw material particle size) were assessed. In the ST, pellet size had a significant effect on relative bead distribution at 16 h post‐feed (*p*  < 0.05). Relative bead distribution 16 h after feeding was significantly higher in fish fed the 3 mm pellet (39.3 ± 8.6%) than the 5 mm pellet (16.6 ± 3.1%). A significant effect of pellet size on relative bead density was also observed in the MI at 24 h (*p*  < 0.05), whilst the relative bead density of the 5 and 3 mm treatments was recorded at 52.7 ± 8.7% and 24.2 ± 5.1%, respectively. There were no significant effects of pellet size (pooling ingredient particle) or raw material particle size (pooling pellet) observed in the DI within time point in this study.

**Table 5:** Analysis of variance tables of GLMs of relative bead densities within gastrointestinal tract regions: ST, MI or DI, for two diet variables: pellet size and raw material particle size over 48 h.

Relative bead density	DF^2^	*F*‐statistic	*p*‐Value
ST			
Time (h)	6	116.382	<0.001
Pellet	1	0.759	0.386
Particle	2	0.230	0.795
Time × pellet	6	2.984	0.011
Time × ingredient particle	12	1.359	0.202
Pellet × ingredient particle	2	0.492	0.613
Time × pellet × ingredient particle	12	0.662	0.782
Residuals	84		
MI			
Time (h)	6	14.097	<0.001
Pellet	1	0.900	0.346
Ingredient particle	2	1.123	0.330
Time × pellet	6	4.124	0.001
Time × ingredient particle	12	1.434	0.167
Pellet × ingredient particle	2	1.948	0.149
Time × pellet × ingredient particle	12	1.853	0.052
Residuals	84		
DI			
Time (h)	6	99.634	<0.001
Pellet	1	2.891	0.093
Ingredient particle	2	2.009	0.140
Time × pellet	6	2.288	0.043
Time × ingredient particle	12	0.951	0.502
Pellet × ingredient particle	2	0.834	0.438
Time × pellet × ingredient particle	12	0.559	0.868
Residuals	84		

**Table 6:** Relative bead distribution (%) in the ST, MI and DI relative to the entire GIT of Atlantic salmon fed one of six dietary treatments: 3 mm: fine, 3 mm: medium, 3 mm: coarse, 5 mm: fine, 5 mm: medium and 5 mm: coarse, assessing the modulation capabilities of two pellet size and three raw material particle sizes for 36 days at 15°C.

Time	Dietary treatment						Ingredient particle			Pellet		*p*‐Value		
	3 mm: fine	3 mm: medium	3 mm: coarse	5 mm: fine	5 mm: medium	5 mm: coarse	Fine	Medium	Coarse	3 mm	5 mm	Pellet (A)	Ingredient particle (B)	A × B
ST														
2	98.4 ± 1.6	91.2 ± 4.7	97.6 ± 2.4	96.1 ± 1.1	84.5 ± 10.2	96.3 ± 3.7	97.3 ± 1.0	87.9 ± 5.2	96.9 ± 2.0	95.8 ± 1.9	92.3 ± 3.7	ns	ns	ns
4	83.8 ± 0.6	83.9 ± 2.7	66.9 ± 15.2	77.5 ± 11.5	80.8 ± 7.2	75.4 ± 8.8	80.7 ± 5.4	82.4 ± 3.5	71.1 ± 8.1	78.2 ± 5.3	77.9 ± 4.7	ns	ns	ns
8	52.2 ± 5.1	63.9 ± 4.3	61.0 ± 7.8	46.1 ± 3.1	63.7 ± 11.6	34.8 ± 6.8	49.2 ± 3.0	63.8 ± 5.5	47.9 ± 7.5	59 ± 3.4.0	48.2 ± 5.8	ns	ns	ns
16	47.4 ± 20.6	28.2 ± 5.2	42.3 ± 18.1	21.6 ± 4.0	6.3 ± 3.6	21.9 ± 2.2	34.5 ± 11.0	17.3 ± 5.7	32.1 ± 9.4	39.3 ± 8.6^a^	16.6 ± 3.1^b^	^∗^	ns	ns
24	3.9 ± 2.7	0.0 ± 0.0	12.1 ± 2	3.3 ± 1.7	29.7 ± 23.7	10.1 ± 7.0	3.6 ± 1.4	14.8 ± 12.5	11.1 ± 3.3	5.3 ± 2.0	14.3 ± 8.2	ns	ns	ns
36	0.0 ± 0.0	6.3 ± 6.3	0.0 ± 0.0	6.7 ± 5.5	21.3 ± 19.4	13.6 ± 7.3	3.4 ± 2.9	13.8 ± 9.7	6.8 ± 4.5	2.1 ± 2.1	13.9 ± 6.5	ns	ns	ns
48	0.0 ± 0.0	3.7 ± 3.7	0.0 ± 0.0	1.6 ± 1.6	0.0 ± 0.0	4.8 ± 4.8	0.8 ± 0.8	1.9 ± 1.9	2.4 ± 2.4	1.2 ± 1.2	2.1 ± 1.6	ns	ns	ns
MI														
2	1.6 ± 1.6	8.8 ± 4.7	2.4 ± 2.4	3.9 ± 1.1	15.5 ± 10.2	3.7 ± 3.7	2.7 ± 1.0	12.1 ± 5.2	3.1 ± 2.0	4.2 ± 1.9	7.7 ± 3.7	ns	ns	ns
4	15.6 ± 0.2	14.6 ± 2.8	27.4 ± 12.9	15.6 ± 7.1	19.2 ± 7.2	11.7 ± 9.3	15.6 ± 3.2	16.9 ± 3.6	19.5 ± 7.9	19.2 ± 4.3	15.5 ± 4.1	ns	ns	ns
8	16.4 ± 7.0	18.6 ± 6.7	13.8 ± 6.9	16.3 ± 7.0	7.8 ± 4.8	27.9 ± 4.6	16.4 ± 4.4	13.2 ± 4.4	20.8 ± 4.9	16.3 ± 3.5	17.3 ± 4.0	ns	ns	ns
16	11.3 ± 4.4	34.2 ± 13.7	20.2 ± 8.7	24.0 ± 4.7	37.1 ± 0.9	42.0 ± 9.5	17.6 ± 4.0	35.7 ± 6.2	31.1 ± 7.6	21.9 ± 5.9	34.4 ± 4.1	ns	ns	ns
24	53.5 ± 6.7^ab^	75.0 ± 14.4^a^	29.6 ± 12.2^ab^	22.0 ± 7.2^b^	15.3 ± 9.7^b^	35.2 ± 7.7^ab^	37.8 ± 8.3	45.1 ± 15.5	32.4 ± 6.6	52.7 ± 8.7^a^	24.2 ± 5.1^b^	^∗^	ns	^∗^
36	22.2 ± 9.5	12.1 ± 6.9	0.0 ± 0.0	25.4 ± 15.2	7.7 ± 7.7	9.4 ± 4.8	23.8 ± 8.0	9.9 ± 4.7	4.7 ± 3.0	11.4 ± 4.7	14.2 ± 5.8	ns	ns	ns
48	4.2 ± 4.2	7.4 ± 7.4	11.1 ± 11.1	0.0 ± 0.0	12.9 ± 10.3	0.0 ± 0.0	2.1 ± 2.1	10.1 ± 5.8	5.6 ± 5.6	7.6 ± 4.2	4.3 ± 3.7	ns	ns	ns
DI														
2	0.0 ± 0.0	0.0 ± 0.0	0.0 ± 0.0	0.0 ± 0.0	0.0 ± 0.0	0.0 ± 0.0	0.0 ± 0.0	0.0 ± 0.0	0.0 ± 0.0	0.0 ± 0.0	0.0 ± 0.0	ns	ns	ns
4	0.6 ± 0.6	1.4 ± 1.4	5.7 ± 4.0	6.9 ± 5.0	0.0 ± 0.0	13.0 ± 11.7	3.7 ± 2.7	0.7 ± 0.7	9.3 ± 5.8	2.6 ± 1.5	6.6 ± 4.1	ns	ns	ns
8	31.4 ± 5.9	17.6 ± 2.4	25.1 ± 2.5	37.6 ± 4.3	28.6 ± 10.7	37.3 ± 6.3	34.5 ± 3.6	23.1 ± 5.5	31.2 ± 4.1	24.7 ± 2.8	34.5 ± 4.1	ns	ns	ns
16	41.3 ± 19.4	37.6 ± 9.0	37.5 ± 12.8	54.4 ± 6.5	56.5 ± 3.4	36.1 ± 9.0	47.9 ± 9.6	47.1 ± 6.0	36.8 ± 7.0	38.8 ± 7.2	49.0 ± 4.7	ns	ns	ns
24	42.5 ± 7.2	25.0 ± 14.4	58.3 ± 10.2	74.7 ± 8.8	55.1 ± 16.0	54.7 ± 11	58.6 ± 8.8	40.0 ± 11.7	56.5 ± 6.8	41.9 ± 7.3	61.5 ± 7.0	ns	ns	ns
36	77.8 ± 9.5	81.5 ± 12.7	100 ± 0.0	67.9 ± 11.6	71.0 ± 17.4	77.0 ± 5.6	72.8 ± 7.1	76.3 ± 9.9	88.5 ± 5.7	86.5 ± 5.7	72.0 ± 6.4	ns	ns	ns
48	95.8 ± 4.2	88.9 ± 11.1	88.9 ± 11.1	98.4 ± 1.6	87.1 ± 10.3	95.2 ± 4.8	97.1 ± 2.1	88.0 ± 6.8	92.1 ± 5.6	91.2 ± 4.8	93.6 ± 3.7	ns	ns	ns

*Note:* Values are expressed as the mean ± SE (*n* = 3, *N* = 18). ANOVA with *T*‐test (pellet) or post hoc Tukey’s test (dietary treatment or ingredient particle). Different superscript letters indicate significant differences (*p* < 0.05) between the means of different treatments.

Abbreviation: ns, not significant.

^∗^Denotes significance (*p* < 0.05).

### 3.5. Estimated Gut Evacuation Time

**Figure 3:**
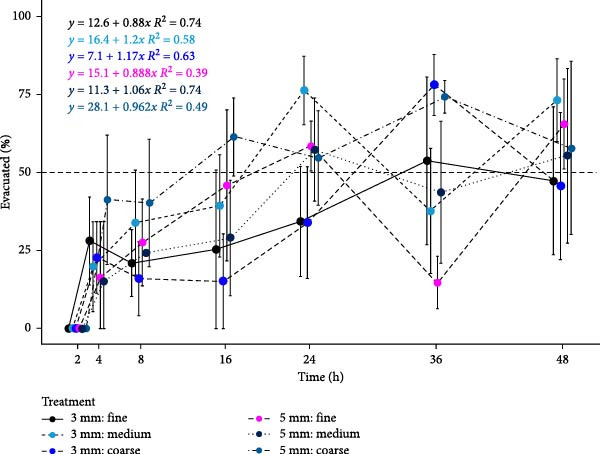
Evacuated feed (%) ± SE (*n* = 3, *N* = 18) from the entire GIT of Atlantic salmon reared at 15°C fed one of six dietary treatments assessing two pellet size and three raw material particle sizes for 36 days. Gut evacuation was quantified by determining the portion of beads that have vacated the GIT relative to gut fullness, being the total number of beads present in the GIT at 2 h post‐feed. The dashed line marks an evacuation level of 50%. Gut evacuation (%) is presented on the *Y*‐axis and time post‐feed (hours) is presented on the *X*‐axis.

Following the determination of relative bead distribution, bead count data was used to quantify the evacuation of feed from the entire GIT (Figure 3). Linear regression analysis was performed for each treatment to plot the evacuation time of feed from the GIT. By solving the linear regression equations, the time at which 50% of the feed had evacuated the GIT (hereto referred to as *T*_50%_) was identified and used to describe differences in transit rate between treatments (Table 7). The treatment that first reached *T*_50%_ was 5 mm: coarse at 22.77 h post‐feed. The final treatment to reach *T*_50%_ was the 3 mm: fine treatment, at 42.50 h.

**Table 7:** Estimated time for fish fed one of six dietary treatments to reach 50% evacuation (*T*_50%_) of the entire GIT (*T*_50% =_ (50% − intercept)/slope).

	Dietary treatment					
	3 mm: fine	3 mm: medium	3 mm: coarse	5 mm: fine	5 mm: medium	5 mm: coarse
*T*_50%_	42.50	28.00	36.67	39.70	36.51	22.81
Intercept	12.60	16.40	7.10	15.10	11.30	28.10
Slope	0.88	1.20	1.17	0.88	1.06	0.96
*r* ^2^	0.74	0.58	0.63	0.39	0.74	0.49

## 4. Discussion

The objective of this study was to explore if physical pellet properties, specifically, pellet size and raw material particle size, can modulate the movement of digesta through the GIT in Atlantic salmon. The information gained through this study may lead to optimised and efficient utilisation of dietary nutrients by providing a better understanding of the effects of GTT in Atlantic salmon. Accelerated GTT is particularly problematic from a nutritional perspective as it limits the time that dietary nutrients are exposed to digestive enzymes, resulting in inefficiencies in nutrient uptake and retention [1]. Importantly, there are clear negative environmental impacts stemming from poor feed efficiency, including an increased prevalence of nutrients being discharged to the surrounding aquatic environment and smothering of benthic communities [18, 22]. These nutritional and environmental concerns subsequently translate into economic loss to the aquaculture industry through inefficient feed use, decreased yield and inefficient nutrient utilisation [1, 8, 18]. However, altering aspects of the diet such as pellet size and raw material particle size to achieve an ideal GTT is within the control of the aquaculture feed industry and relatively straightforward to implement on farm. Coupling these issues of inefficient feed utilisation with the unwavering climate challenges now facing the global aquaculture industry highlights the immediate need to mitigate the digestive response to thermal stress [34]. To date, various studies have assessed pellet size and raw material particle size to determine if they had any detectable effect of GTT in various cultured species [14, 15, 25]. The research described herein focused on the manipulation of diet parameters to modulate GTT with the view of improving nutrient uptake in fish. The absence of targeted research addressing GTT and decreases in nutrient utilisation in cultured Atlantic salmon warrants the need for such a study.

Findings identified that the combined effect of pellet and raw material particle size (treatment) resulted in few significant effects in GTT of Atlantic salmon. In the MI at 24 h post‐feed, relative bead density was greatest in the 3 mm: medium treatment group. A high relative bead density in the MI in the later stages of the trial would suggest minimal gastric emptying, and therefore, a slower evacuation time. Therefore, based on the results obtained at 24 h post‐feeding, the 3 mm: medium treatment appears to exhibit the slowest transit. However, a smaller pellet size has been associated with faster transit rate in comparison to a larger pellet in past literature [15]. Data obtained by Busti et al. [15] assessing the relationship between the singular variable of pellet size on the GTT of gilthead sea bream determined that fish fed a 6 mm pellet elicited a slower GTT than fish fed a 2 or 4 mm pellet. While this particular significant relationship between the 3 mm: medium treatment and GTT herein does not appear to directly align with the works of Busti et al. [15], past studies assessing the movement of digesta through the MI have concluded that data obtained from this region is inherently variable, which may in part explain the observed discrepancies between the bodies of work [28, 35]. This could be attributable to the inherent challenges associated with drawing meaningful conclusions from the MI data. While this portion of the GIT is important for enzymatic activity and nutrient absorption, variable trends observed in past work suggest that the MI serves as a middle ground between the ST and DI which are essential in the breakdown and evacuation of ingested feeds, respectively [3, 5]. Similar to pellet size, raw material particle size has been positively linked to transit time, with a coarser particle size resulting in slower gut evacuation [14]. This is likely correlated to the large surface‐to‐volume ratio of smaller particles which facilitates a rapid mechanical breakdown, resulting in a faster GTT than observed in the smaller raw material particle size diets [23, 26]. When assessing gastric emptying in Atlantic salmon, Sveier et al. [14] concluded that gut evacuation was slower in fish fed a coarser raw material particle size (3000–5000 μm) than those fed a pellet containing a finer (100–300 μm) or standard (<1000 μm) grind size. The comparatively slower GTT observed in the MI in fish fed the 3 mm: medium diet somewhat align with the findings presented by Sveier et al. [14], but not across all treatments. The absence of clear trends may be attributed to the influence of the pellet size variable, or simply because of the variability that is synonymous with the MI [3, 5]. Aside from the significant relationships between treatment and the relative bead density present in the ST and MI, the data obtained in this study suggest that the combined effects of the assessed variables in general, did not result in any clear trends in GTT in Atlantic salmon.

Following the examination of the effect of the six dietary treatments, pellet (pooling raw material particle size) and raw material particle size (pooling pellet size) were examined independently. Analysis of pellet size determined that relative bead density significantly influenced GTT in the ST and MI at 16 and 24 h post‐feed, respectively. At these time points, relative bead density of the ST and MI was higher in fish fed the 3 mm pellet. A high bead count in the ST region in the early stages of the trial would indicate slower transit, conflicting with past studies stating that a larger pellet size was associated with slower GTT in gilthead seabream [15]. Similarly, data obtained from the DI suggests a faster transit time in the 5 mm pellet in comparison to the 3 mm pellet. Slower gut transit in fish fed larger pellets has been attributed to the longer time period required to adequately moisturise and subsequently disintegrate via mechanical digestion directly following ingestion [15]. Mechanical digestion refers to the physical disintegration of ingesta into smaller units, which is more laborious for a meal with a lower surface area to volume ratio [25, 36]. This results in less exposure to processes such as mastication and peristalsis that impact the breakdown of feed [37, 38]. While data drawn from the ST in this study between 2 and 16 h post‐feed (inclusive) suggest that a faster transit time in response to the larger pellet size, GTT tended to be slower in the 5 mm treatment in the sampling points that followed, aligning with the past works [15]. However, by this point in the study over 50% of the digesta had evacuated the ST and released into subsequent digestive tract regions for nutrient absorption. Despite a statistically higher relative bead density in response to the 3 mm pellet at 24 h, the MI appeared to be void of any clear trends in relation to pellet size. Results obtained from the individual assessment of pellet size and raw material particle size showed few significant effects on GTT, suggesting that these variables are unlikely to have strong modulatory capabilities.

To complement the GTT data, as assessed by relative bead density, the evacuation time (%) of the entire GIT in response to dietary treatment was determined by quantifying the number of ingested beads that had left the GIT at each time point. An evacuation time of 50% (*T*_50%_) was selected as a useful point of comparison between treatments as this can be considered the ‘tipping point’ at which the majority of feed in the GIT has been excreted. The first treatment to record a 50% evacuation of feed from the GIT was the 5 mm: coarse diet at 22.8 h post‐feeding. In contrast, the 3 mm: fine treatment, exhibited the slowest evacuation time reaching *T*_50%_ at 42.5 h. Despite the absence of clear differences between treatments when examining the linear regression plots, the calculations of *T*_50%_, that is, the time for 50% of the feed to leave the GIT, generally agreed with the trends in relative bead density. While there was no statistically significant effect of raw material particle size on feed transit between GIT regions when considering the relative distribution of feed between the GIT regions, the assessment of total gut evacuation, in particular the calculation of *T*_50%_, depicted a faster evacuation of feed in fish fed a diet with a larger raw material particle size compared to those manufactured using raw materials with finer grind size. This result presents new insights for Atlantic salmon that differ somewhat with some previous studies where a finer raw material particle size resulted in a faster gut evacuation time [14, 15]. To further explore this result, other physical properties of the pellet, namely, pellet hardness and bulk density were assessed. In the present study, pellet hardness increased with decreasing grind size. Previous research exploring the relationship between pellet hardness following soaking or subjected to altered extrusion parameters and gastric function, found that gut evacuation could be impaired by harder pellets [37, 39, 40]. Therefore, the faster transit time of feed with finer ingredient particles in the present study may be attributed to the associated increase in pellet hardness and not solely due to the grind size of the raw materials used. It should be noted that the parameters of the aforementioned studies consider variables other than pellet or raw material particle size, and thus, direct comparisons are limited [37, 39, 40]. In addition to pellet hardness, gastric emptying has also been correlated to the density of feed pellets [41], where in the present study, feeds with finer ingredient particles tended to have a greater bulk density, which has previously been associated with a slower transit time [41–43]. This may, in‐part, be due to an increase in viscosity as raw material particle size decreases, thereby inhibiting the transit of fine ingredient particles through the GIT [44]. Therefore, it appears that raw material particle size and, importantly, the association with raw material particle size and pellet hardness and density, assert notable influence of feed evacuation time in Atlantic salmon.

Similar to past studies investigating GTT in fish, the data herein presented a clear negative relationship between relative bead density and time in the ST, and conversely, a positive relationship in the DI [3]. Data exhibited the expected progression of feed digesta through the complete GIT, consistent with past reports [3]. Additionally, across all treatments, the time point at which ST fullness first decreases below 50%, DI fullness will reach 50% of its maximum relative bead density. Alike the *T*_50%_ value, this is the point at which the majority of feed in the GIT has passed mechanical digestive processes and has entered a primarily enzymatic phase of intestinal digestion. This relationship can be described using the following formula:

Where SRbtx>50, DRbtx<D max2,

where *tx* is the time point of event, SRb is the proportion of feed (%) in the ST at *t*, DRb is the proportion of feed (%) in the DI at *t*, *D*_max_ is the maximum proportion of feed (%) observed in the DI across all *t* events.

Alongside the overarching trend of transit from the ST region to the DI overtime, a stepwise trend was observed in ST emptying overtime, demonstrating the periodic release of digesta to the MI. The pulse emptying model proposed by Jobling [25] suggests that the muscular contractions of the ST wall and the pyloric sphincter are not continuous, resulting in a non‐linear relationship between time and ST content, which was measured in this study as relative bead density. Furthermore, Jobling [25] identified that the sampling time points in GTT studies are often spread out over multiple days, meaning that discrete changes in ST fullness, even MI and DI capacity, are not captured. While some minor trends in GTT are missed using a time‐point sampling technique, as utilised in this study, sampling points in the early stage of the sampling phase were relatively frequent, capturing both the periodic release for digesta from the ST to the MI, and the overall trend of digesta transitioning through the regions of interest. Therefore, this study, among others, attest to the robustness of this sampling design in determining differences in the GTT of fish subjected to experimental variables [3, 28].

In addition to investigating GTT, the apparent digestibility of the diets was assessed to potentially identify effects of pellet and raw material particle size. There were no statistically significant differences between the ADC values of the treatments. However, all aspects of ADC, were numerically higher in the 3 mm: fine pellet. An assessment of pooling pellet and raw material particle size found that dry matter and gross energy digestibility was significantly greater in fish fed the fine particle size (pooling pellet). Additionally, protein ADC was statistically higher in fish fed the 3 mm pellet (pooling raw material particle size), although the values only varied by 0.8%. It is likely that the observed difference is caused by analytical variability. However, smaller pellet size may allow for efficient absorption despite a generally faster transit time. Khater et al. [45] found that there was a positive relationship between pellet diameter and pellet durability, meaning that a greater amount of force was required to disintegrate pellets of a larger size. Assessment of pellet hardness in this study supported this notion, suggesting that the 5 mm pellet could withstand a greater amount of force prior to disintegration. Therefore, it stands to reason that the 3 mm pellet assessed could facilitate prompt enzymatic activity and the subsequent nutrient retention. The fine particle size used in the 3 mm: fine treatment may have also contributed to the generally higher digestibility observed in this treatment compared to the other experimental diets. A pellet formed using a fine particle size has a greater surface‐to‐volume ratio, hence greater area for potential exposure to digestive enzymes [26]. The efficiency of the chemical portion of the digestive process, much like mechanical digestion, can be informed by the surface area of the food source [4, 46]. The capacity for digestive enzymes present in the gut such as protease, lipase and amylase, to hydrolyse dietary nutrients is positively related to the area of the substrate [36, 47]. Therefore, smaller raw material particle sizes in aquafeed appear to have a positive influence on nutrient assimilation in fish [25, 47]. In the present study, diets of a smaller pellet and raw material particle size marginally improve nutrient digestibility in comparison to diets consisting of coarser raw materials and a larger pellet diameter. While numerically minor, these can translate to substantial improvements in feed efficiency when applied to large scale farming practices. However, nutrient digestibility did not consistently correlate to GTT, suggesting that the physical properties of feed may exert a large influence on nutrient digestibility in fish rather than GTT alone. Yet, given the intuitive interrelatedness of GTT and pellet physical properties, further studies are recommended with a view of isolating the drivers of the digestive function and efficiency.

## 5. Conclusion

Findings generated by this study have determined that physical pellet properties can, to a degree, influence the transit of digesta in cultured Atlantic salmon. Specifically, diets of varying pellet size or raw material particle size were shown to alter the time required for at least 50% of the ingested meal to pass through the GIT, whereby the smallest, finest diet was retained for the longest period. Whilst the combination of pellet and raw material particle sizes generated few significant outcomes with respect to GTT, it was determined that larger pellet and raw material particle sizes resulted in a more rapid gut evacuation time compared to the diets of a smaller pellet and finer ingredient particle grind size. As such, this study will better inform, at least in part, the necessary physical properties of commercial diets developed for cultured Atlantic salmon exhibiting rapid GTT in response to novel dietary ingredients or temperature. In turn, this will improve the utilisation of feed by fish, reducing the quantity of feed required to reach commercial growth targets, subsequently limiting the production of waste produced. Combined, each of these factors will contribute to the economic and environmental sustainability of the aquaculture industry.

## Conflicts of Interest

The authors wish to declare the donation of materials for the growth trial from Ridley AgriProducts Pty Ltd (Narangba, Queensland, Australia). The authors wish to clarify that the current trial did not directly compare products commercially produced by Ridley AgriProducts.

## Funding

This study was funded by the Cooperative Research Centres Program (Grant GA72953).

## Data Availability

The data that support the findings of this study are available from the corresponding author upon reasonable request.
